# Mitochondrial acclimation potential to ocean acidification and warming of Polar cod (*Boreogadus saida*) and Atlantic cod (*Gadus morhua*)

**DOI:** 10.1186/s12983-017-0205-1

**Published:** 2017-04-14

**Authors:** Elettra Leo, Kristina L. Kunz, Matthias Schmidt, Daniela Storch, Hans-O. Pörtner, Felix C. Mark

**Affiliations:** 1 Alfred Wegener Institute, Helmholtz Centre for Polar and Marine Research, Integrative Ecophysiology, Am Handelshafen 12, D-27570 Bremerhaven, Germany; 2 University of Bremen, Fachbereich 2, NW 2/Leobener Strasse, D-28359 Bremen, Germany; 3 Alfred Wegener Institute, Helmholtz Centre for Polar and Marine Research, Bentho-Pelagic Processes, Am Alten Hafen 26, D-27568 Bremerhaven, Germany

**Keywords:** Arctic fish, RCP 8.5, Heart mitochondria, Mitochondrial capacity, Proton leak

## Abstract

**Background:**

Ocean acidification and warming are happening fast in the Arctic but little is known about the effects of ocean acidification and warming on the physiological performance and survival of Arctic fish.

**Results:**

In this study we investigated the metabolic background of performance through analyses of cardiac mitochondrial function in response to control and elevated water temperatures and *P*CO_2_ of two gadoid fish species, Polar cod (*Boreogadus saida*), an endemic Arctic species, and Atlantic cod (*Gadus morhua*), which is a temperate to cold eurytherm and currently expanding into Arctic waters in the wake of ocean warming. We studied their responses to the above-mentioned drivers and their acclimation potential through analysing the cardiac mitochondrial function in permeabilised cardiac muscle fibres after 4 months of incubation at different temperatures (Polar cod: 0, 3, 6, 8 °C and Atlantic cod: 3, 8, 12, 16 °C), combined with exposure to present (400μatm) and year 2100 (1170μatm) levels of CO_2_.

OXPHOS, proton leak and ATP production efficiency in Polar cod were similar in the groups acclimated at 400μatm and 1170μatm of CO_2_, while incubation at 8 °C evoked increased proton leak resulting in decreased ATP production efficiency and decreased Complex IV capacity. In contrast, OXPHOS of Atlantic cod increased with temperature without compromising the ATP production efficiency, whereas the combination of high temperature and high *P*CO_2_ depressed OXPHOS and ATP production efficiency.

**Conclusions:**

Polar cod mitochondrial efficiency decreased at 8 °C while Atlantic cod mitochondria were more resilient to elevated temperature; however, this resilience was constrained by high *P*CO_2_. In line with its lower habitat temperature and higher degree of stenothermy, Polar cod has a lower acclimation potential to warming than Atlantic cod.

## Background

Ocean warming driven by anthropogenic CO_2_ emissions influences the distribution of marine animals causing significant impacts on biodiversity and ecosystem structure [1, 2], such as local extinctions [3] and poleward migrations [4–6]. Fish (and other ectotherms) are particularly sensitive to fluctuations in temperature since their body temperature is in equilibrium with their environmental temperature [7]. Fish species distribution, in fact, is confined to a specific temperature window, due to the temperature dependency of physiological processes and to sustain maximal energy efficiency ([8] for review).

The increased CO_2_ concentration in the atmosphere is one of the major causes for the global greenhouse effect and also causes a decrease in ocean pH, a phenomenon commonly known as ocean acidification [9]. High CO_2_ partial pressure (*P*CO_2_) is known to affect biological and physiological processes of marine organisms (e.g. [10–14]) and tolerances towards other stressors [15–17]. Moreover, high *P*CO_2_ could provoke a narrowing of the thermal tolerance window of ectotherms, so that limits of its thermal acclimation capacity are met earlier [2, 18–21].

At the cellular level, exposure to high temperature can cause changes in the three dimensional structures of proteins, including the assembly states of multiprotein complexes and eventually protein denaturation and loss of activity [7]. Moreover, increasing temperatures can alter the cellular membranes packing order, which can cause changes in membrane-associated processes until a potential complete loss of function [22]. Furthermore, since cellular oxygen demand increases with increasing temperature, the production of mitochondrial reactive oxygen species (ROS) is likely to increase which can damage biological molecules, including lipids, proteins and DNA [23, 24]. Therefore, towards the upper limit of the thermal window, the cellular energetic costs for maintenance rise, increasing baseline energy turnover and allowing only for time-limited periods of passive tolerance. If high temperature persists over this period of passive tolerance, the costs of maintenance can only be covered at the expense of other functions such as growth and reproduction, decreasing the overall animal fitness [17]. Therefore, in light of ongoing ocean acidification and warming it is important to understand how fish respond to increasing habitat temperatures, their ability to adjust their thermal sensitivity and the role that high *P*CO_2_ plays in thermal acclimation [2, 25].

The fish heart is highly aerobic and sensitive to temperature [26, 27]. Its capacity limits have been hypothesized to shape the warming-induced onset of sublethal thermal constraints in fishes [2, 28–31]. Recent studies have shown that high temperature leads to heart failure in various fish species like New Zealand triplefins and temperate and tropical wrasses [28, 29, 32, 33]. It was suggested that progressive impairment of several components of the mitochondrial function measured in permeabilised heart muscle fibres, such as oxidative phosphorylation (OXPHOS, respiratory state III), ATP production efficiency and the capacity of single complexes of the Electron Transport System (ETS) shape the temperature of heart failure (T_HF_). High temperature changes the fluidity of mitochondrial membranes, which can entail increased proton leak through the inner membrane ([19] for review), resulting in decreased coupling ratios and causing decreased membrane potential [34, 35] and, as a consequence, inhibit the electrogenic transport of substrates, i. e. the transport of charged substrates like glutamate and malate that leads to the translocation of net charge across the membrane [36]. This indicates that mitochondrial metabolism is involved in functional constraints and thermal limitation of this tissue [28, 29, 32, 33]. Therefore, alterations in cardiac mitochondrial metabolism might lead to impaired cardiac energy turnover and, as a consequence, constraints in cardiac performance and ultimately affect the fishes’ thermal sensitivity.

Although an extensive literature has been produced on the effects of temperature on fish cellular metabolism and mitochondrial function (e.g. [8, 33, 37] and the literature therein), only few studies have addressed the effects of moderately elevated *P*CO_2_ on them [30, 38–41]. Moreover, as ocean warming and ocean acidification caused by high *P*CO_2_ are two sides of the same coin, they must be considered in combination in order to draw ecologically realistic conclusions [17, 42, 43].

Ocean acidification and warming trends are projected to exert particularly strong effects in the Arctic. As one of the consequences, temperate species may become established in Arctic habitats (by poleward migration), potentially displacing resident taxa [1, 4, 6]. For example, in the past decade the Northeast Arctic population of Atlantic cod (*Gadus morhua,* NEAC) has expanded its range into the Barents Sea [44, 45], on the North-east Greenland shelf [46] and in the coastal waters around Svalbard, which are inhabited by native Polar cod (*Boreogadus saida*), a key species in this region [1, 47].

Polar cod is a permanently cold adapted Arctic fish (thermal habitat around Svalbard ranging from −2 to +7 °C [48, 49]) while NEAC is a cold acclimated sub-Arctic population of temperate Atlantic cod expanding into the Arctic (habitat thermal range around Svalbard: 0–8 °C [1, 50]). Cold-acclimated and -adapted fish are known to have elevated mitochondrial densities. Among cold adapted species, extreme stenotherms such as high Antarctic fish, have high densities but low mitochondrial capacities and low proton leak in aerobic tissues [37, 51–53]. This may result in the low maintenance costs derived by proton leak and narrow thermal windows of these species and, as a consequence, cause high sensitivity to ocean warming [53, 54]. On the other hand, eurythermal cold adaptation ensures mitochondrial function over a wider range of temperatures at lower mitochondrial densities and maximized capacities [53, 55]. As a permanently cold adapted fish, Polar cod may therefore not be able to adjust mitochondrial capacities during warming to a similar extent as NEAC, which apparently has a higher capacity to adjust to higher temperatures by decreasing mitochondrial densities and capacities and thereby developing the metabolic plasticity necessary to acclimate to new conditions [56]. The differences in thermal response and, in particular, the ability to acclimate to higher temperatures will play a central role for their interaction in a changing ecosystem.

Hence, the aim of this study was to investigate the acclimation potential of Polar cod *Boreogadus saida* and Northeast Arctic cod (NEAC) *Gadus morhua* exposed to water temperatures and *P*CO_2_ projected for the year 2100 in the Arctic i.e. 8 °C and 1170μatm *P*CO_2_ (RCP 8.5 [57]). For a deeper understanding of the impact of ocean acidification and warming on the bioenergetics of the two species in relation to thermal tolerance, we further investigated mitochondrial function in the cardiac muscle of animals incubated for 4 months at four different temperatures (Polar cod: 0, 3, 6, 8 °C and Atlantic cod: 3, 8, 12, 16 °C), and two *P*CO_2_ (400μatm and 1170μatm) in a cross factorial design. We used permeabilised cardiac muscle fibres to investigate a system resembling the living state as closely as possible [58–60], facilitating the extrapolation from measurements of cardiac mitochondrial capacities to their potential effects on the heart and eventually drawing conclusions on the effects of high temperature and high *P*CO_2_ on the whole organism. Moreover, by analysing the mitochondrial function at the respective incubation temperature we could investigate the acclimation potential of the two species. We hypothesized that NEAC had higher thermal limits and a larger acclimation capacity than Polar cod and found accordingly that mitochondrial functions are constrained at lower temperatures in Polar cod than in NEAC. We discuss our results in light of the findings reported by Kunz et al. [61], who showed wider thermal windows for growth and standard metabolic rate (SMR) in NEAC than in Polar cod from the same acclimation experiment.

## Methods

### Animal collection

Juvenile Polar cod were collected by bottom trawl in combination with a fish lift [62] on January 17^th^, 2013 from the inner part of Kongsfjorden (Svalbard, 78° 97’ N 12°51’ E) at 120 m depth and a water temperature between 2 and 3 °C. They were kept at 3.3–3.8 °C in the facilities of the Tromsø Aquaculture Research Station, in Kårvik (Norway) until late April 2013 when they were transported to the aquarium facilities of the Alfred Wegener Institute (AWI) in Bremerhaven (Germany), where they were kept at 5 °C, 32 PSU and ambient *P*CO_2_ until the start of the incubation.

Juvenile Northeast Arctic cod (NEAC) were caught in late August 2013 in several locations off Western Svalbard during RV Heincke cruise HE408 in Rijpfjorden (80° 15.42' N 22° 12.89' E), Hinlopenstretet (79° 30.19' N 18° 57.51' E), and Forlandsundet (78° 54.60' N 11° 3.66' E) at 0–40 m depth and water temperatures between 3.5 and 5.5 °C using a pelagic midwater trawl in combination with a fish lift [62]. The specimens were transported to the AWI facilities in Bremerhaven (Germany), where they were kept at 5 °C, 32 PSU and ambient *P*CO_2_ until the start of the incubation.

### Incubation

Polar cod incubation started in June 2013 and of NEAC in May 2014. After at least 4 weeks of acclimation to laboratory conditions (5 °C, 32 PSU and ambient *P*CO_2_), individuals from both species were housed in single tanks and randomly allocated to the temperature and *P*CO_2_ incubation set-up with a 12 h day/night rhythm. The respective *P*CO_2_ conditions were pre-adjusted in a header tank containing ~200 l of seawater. Virtually CO_2_-free pressurized air and pure CO_2_ were mixed by means of mass flow controllers (4 and 6 channel MFC system, HTK, Hamburg, Germany) to achieve the desired *P*CO_2_. Temperature was adjusted by 1 °C per day for each group starting from 5 °C. *P*CO_2_ in the high *P*CO_2_ group was adjusted within 1 day after the incubation temperature was reached. The animals were kept under incubation conditions for 4 months and fed *ad libitum* with commercial pellet feed (Amber Neptun, 5 mm, Skretting AS, Norway) every fourth day [61]. The sampling of Polar cod and NEAC took place after 4 days of fasting, due to sampling and experimental logistics three to six individuals of Polar cod and four to eight individuals of NEAC were sampled in one batch. Because of a failure in the power supply the group incubated at 3 °C and high *P*CO_2_ died before the mitochondrial capacity could be investigated.

Average length and weight, as well as the number of the specimens per treatment at the time of sampling are given in Table 1.

**Table 1:** Total length, body weight and number of fish (*n*) used for testing cardiac mitochondrial respiration in Polar cod (*B. saida*) and NEAC (*G. morhua*)

Acclimation	Species					
	*B. saida*			*G. morhua*		
	Total length (cm)	Body weight (g)	*n*	Total length (cm)	Body weight (g)	*n*
0 °C control	15.28 ± 0.37	22.88 ± 2.05	5	-	-	-
0 °C high	14.30 ± 0.64	19.22 ± 2.61	6	-	-	-
3 °C control	15.62 ± 0.98	27.16 ± 6.25	3	20.04 ± 0.92	60.84 ± 9.81	5
3 °C high	-	-	-	21.61 ± 0.46	78.19 ± 6.91	8
6 °C control	15.73 ± 0.21	25.21 ± 1.14	6	-	-	-
6 °C high	17.52 ± 0.61	32.17 ± 2.90	5	-	-	-
8 °C control	15.18 ± 0.72	20.52 ± 2.56	6	23.26 ± 1.75	99.04 ± 22.13	5
8 °C high	15.07 ± 0.47	18.76 ± 1.11	4	21.51 ± 0.82	80.51 ± 10.46	8
12 °C control	-	-	-	22.70 ± 0.80	98.70 ± 13.14	6
12 °C high	-	-	-	23.42 ± 0.72	100.75 ± 9.22	8
16 °C control	-	-	-	21.56 ± 0.69	81.48 ± 9.37	4
16 °C high	-	-	-	24.27 ± 1.91	133.13 ± 31.87	6

“control” and “high” indicate control (400μatm) and high (1170μatm) CO_2_ concentrations. Values are given as means ± S.E.M

### CO_2_ and carbonate chemistry

Temperature, salinity, DIC and pH (total scale) were measured once to twice a week in triplicates in order to monitor the seawater chemistry of the incubation. Temperature and salinity were measured with a WTW LF 197 multimeter (WTW, Weilheim, Germany). pH was measured with a pH meter (pH 3310, WTW, Weilheim, Germany) calibrated with thermally equilibrated NBS-buffers (2-point-calibration). The pH-values were then corrected to pH Total scale using pH-defined Tris-Buffer (Batch 4, Marine Physical Laboratory, University of California, San Diego, CA, USA).

DIC was measured by a Seal QuAAtro SFA Analyzer (800 TM, Seal Analytical, Mequon, United States of America). Calculations of the carbonate system were conducted using CO2sys [63], applying the K1, K2 constants after Mehrbach et al. [64], refitted after Dickson and Millero [65] and using KHSO4 dissociation constants after Dickson [66] assuming a pressure of 10 dbar.

Complete summaries of the seawater parameters and raw data for both species are available from the Open Access library PANGAEA [67, 68].

### Preparation of permeabilised cardiac fibres

Fish were anaesthetized with 0.2 g l^−1^ tricaine methane sulphonate (MS222) and killed by a spinal cut behind the head plate. Hearts were rapidly excised and washed with ice-cold modified relaxing buffer BIOPS (2.77 mM CaK_2_EGTA, 7.23 mM K_2_EGTA, 5.77 mM Na_2_ATP, 6.56 mM MgCl_2_, 20 mM taurine, 15 mM Na_2_-phosphocreatine, 20 mM imidazole, 0.5 mM dithiothreitol, 50 mM MES, 220 mM sucrose, pH 7.4, 380 mOsmol l^−1^; modified after [69]). Hearts were then separated in fibres and placed in 2 ml ice-cold BIOPS containing 50 μg ml^−1^ saponin and gently shaken on ice for 20 min. Fibres were then washed three times for 10 min in 2 ml ice-cold modified mitochondrial respiration medium MIR05 (0.5 mM EGTA, 3 mM MgCl_2_, 60 mM K-lactobionate, 20 mM taurine, 10 mM KH_2_PO_4_, 20 mM HEPES, 160 mM sucrose, 1 g l^−1^ bovine albumine serum, pH 7.4, 380 mOsmol l^−1^) [29, 69].

Directly before experimentation, a subsample of about 10 mg fibres was blotted dry, weighed and introduced into the oxygraph sample chambers.

### Mitochondrial respiration

Mitochondrial respiration was recorded using Oroboros Oxygraph-2 k™ respirometers (Oroboros Instruments, Innsbruck, Austria) and measured as weight-specific oxygen flux [pmol O_2_ (mg fresh weight sec)^−1^] calculated in real time using Oroboros DatLab Software 5.2.1.51 (Oroboros Instruments, Innsbruck, Austria).

All analyses were performed at the respective incubation temperatures, with *c*O_2_ in a range from ~370 nmol ml^−1^ (100% air saturation) to 100 nmol ml^−1^ and *P*CO_2_ at atmospheric levels.

A substrate-uncoupler-inhibitor titration (SUIT) protocol was used on the permeabilised cardiac fibres to investigate the partial contributions of the single components of the phosphorylation system [69]). NADH - Coenzyme Q oxidoreductase (Complex I, CI) and Succinate dehydrogenase (Complex II, CII) substrates (10 mM glutamate, 2 mM malate, 10 mM pyruvate and 10 mM succinate) were added. Saturating ADP (3 mM) was added to stimulate oxidative phosphorylation (OXPHOS). Cytochrome c (10 μM) was added to test the integrity of the outer membrane. Respiration state IV^+^ was measured by addition of atractyloside (0.75 mM) or oligomycin (6 μM) (for Polar cod and NEAC respectively) and step-wise (1 μM each) titration of carbonyl cyanide *p*-(trifluoromethoxy) phenyl-hydrazone (FCCP) was used to uncouple mitochondria (ETS). Complex I, Complex II and Coenzyme Q – cytochrome c reductase (Complex III, CIII) were inhibited by the addition of rotenone (0.5 μM), malonate (5 mM) and antimycin a (2.5 μM), respectively. Lastly the activity of the Cytochrome c oxidase (Complex IV, CIV) was measured by the addition of the electron donor couple ascorbate (2 mM) and *N,N,N* ^*1*^ *,N* ^*1*^-tetramethyl-*p*-phenylenediamine (TMPD, 0.5 mM).

All chemicals were obtained from Sigma-Aldrich (Germany).

### Data analysis

Mitochondrial respiration rates are expressed per mg fresh weight of cardiac fibres and the values are given as means ± S.E.M. OXPHOS coupling efficiency was calculated as [(OXPHOS-State IV^+^) OXPHOS^−1^] after Gnaiger [70].

Normal distribution of the data was assessed by Shapiro-Wilk test and homoscedasticity was evaluated by F-test or Bartlett test in case of two or more groups, respectively. Differences between *P*CO_2_ treatments within the same temperature treatment were evaluated by Student’s *t*-test (with Welch’s correction in case of non-homoscedastic data). Differences across temperatures in the same *P*CO_2_ treatment were evaluated with one-way ANOVA followed by Tukey’s test for the comparison of means.

The level of statistical significance was set at *p* <0.05 for all the statistical tests.

All statistical tests were performed using R 3.2.0 and the “stats” package [71].

## Results

**Fig. 1:**
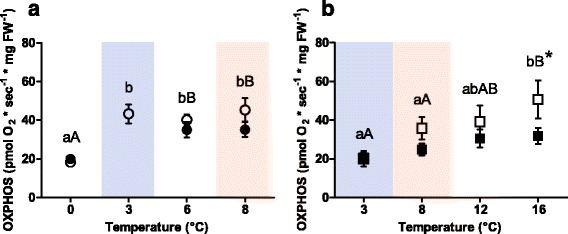
Maximal oxidative phosphorylation capacity (OXPHOS) of permeabilised heart muscle fibres of (**a**) Polar cod (*B. saida*) and (**b**) NEAC (*G. morhua*). Different letters within panels indicate significant differences (*p* <0.05) between temperature treatments; *lower case letters*: control *P*CO_2_ (400μatm), *upper case letters*: high *P*CO_2_ (1170μatm), * indicates significant differences (*p* <0.05) between CO_2_ groups at the same temperature. All values are reported as means ± S.E.M. (for *n* refer to Table 1). *Open symbols*: control *P*CO_2_ (400μatm), *filled symbols*: high *P*CO_2_ (1170μatm). *Circles*: Polar cod, *Squares*: NEAC. *Blue box*: cold shared incubation temperature (3 °C), *Red box*: warm shared incubation temperature (8 °C) between the two species

The maximal oxidative phosphorylation capacity (OXPHOS) of permeabilised heart fibres of both species is shown in Fig. 1. In Polar cod, the groups incubated under control *P*CO_2_ showed significantly lower OXPHOS flux in the 0 °C acclimated fish than in all further incubation groups (3 °C, *p* = 0.007; 6 °C, *p* = 0.007; 8 °C, *p* = 0.001). Mitochondrial respiration was at a similar level in the groups incubated at 3, 6 and 8 °C (*p* >0.05). High *P*CO_2_ levels did not affect OXPHOS, with no differences between the OXPHOS of the groups incubated at the two *P*CO_2_ levels within a temperature treatment (*p* >0.05). The groups incubated under high *P*CO_2_ displayed fluxes that were similar at 6 and 8 °C (*p* >0.05) but significantly higher than in the 0 °C incubated group (*p* = 0.04, Fig. 1a).

Temperature had a significant effect on the OXPHOS of NEAC, with fluxes increasing with incubation temperature (control *P*CO_2_: F = 4.74, *p* = 0.02; high *P*CO_2_: F = 3.78; *p* = 0.02, Fig. 1b). Moreover, the 16 °C/high *P*CO_2_ incubated group showed a lower OXPHOS compared to the 16 °C/control *P*CO_2_ group (*p* = 0.03). This resulted in a more evident plateauing of OXPHOS between 12 and 16 °C in the group incubated under high *P*CO_2_. Comparing the two species, Polar cod had significantly higher OXPHOS capacities than NEAC at both 3 °C (*p* = 0.01, Fig. 1 blue box) and 8 °C (control *P*CO_2_: *p* = 0.04; high *P*CO_2_: *p* = 0.04, Fig. 1 red box).

**Fig. 2:**
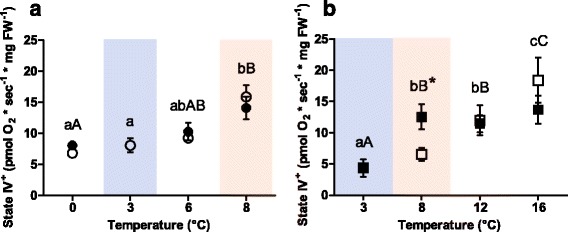
State IV^*^ of permeabilised heart muscle fibres of (**a**) Polar cod (*B. saida*) and (**b**) NEAC (*G. morhua*). Different letters within panels indicate significant differences (*p* <0.05) between temperature treatments; *lower case letters*: control *P*CO_2_ (400μatm), *upper case letters*: high *P*CO_2_ (1170μatm), * indicates significant differences (*p* <0.05) between CO_2_ groups at the same temperature. All values are reported as means ± S.E.M. (for *n* refer to Table 1). *Open symbols*: control *P*CO_2_ (400μatm), *filled symbols*: high *P*CO_2_ (1170μatm). *Circles*: Polar cod, *Squares*: NEAC. *Blue box*: cold shared incubation temperature (3 °C), *Red box*: warm shared incubation temperature (8 °C) between the two species

**Fig. 3:**
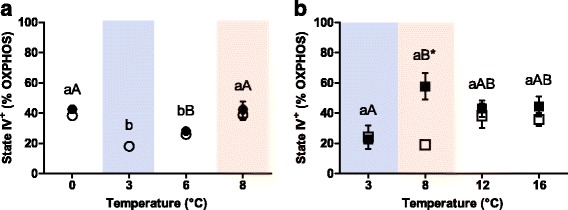
Percentage of oxygen consumed by State IV^+^ in relation to OXPHOS in permeabilised heart muscle fibres of Polar cod (*B. saida,* panel **a**) and NEAC (*G. morhua,* panel **b**). Different letters within the panels indicate significant differences (*p* <0.05) between temperature treatments; *lower case letters*: control *P*CO_2_ (400μatm), *upper case letters*: high *P*CO_2_ (1170μatm), * indicates significant differences (*p* <0.05) between CO_2_ groups at the same temperature. All values are reported as means ± S.E.M. (for *n* refer to Table 1). *Open symbols*: control *P*CO_2_ (400μatm), *filled symbols*: high *P*CO_2_ (1170μatm). *Circles*: Polar cod, *Squares*: NEAC. *Blue box*: cold shared incubation temperature (3 °C), *Red box*: warm shared incubation temperature (8 °C) between the two species

In both species, state IV^+^ was sensitive to temperature (Fig. 2): in Polar cod it remained unchanged in the groups incubated at 0, 3 and 6 °C (*p* >0.05) but was significantly higher in animals incubated at 8 °C compared to the other incubation groups (6 to 8 °C/control *P*CO_2_: *p* = 0.01; 6 to 8 °C/high *P*CO_2_: *p* = 0.04) as shown in Fig. 2a. Quantifying State IV^+^ as a percent fraction of OXPHOS, it was close to 20% and thus lowest in the 3 °C and 6 °C groups of *B. saida*, while at 0 and 8 °C the fraction of State IV^+^ exceeded these values about two-fold as shown in Fig. 3.

In NEAC, State IV^+^ increased along with incubation temperature (control *P*CO_2_: F = 5.96; *p* = 0.02, high *P*CO_2_: F = 12.43; *p* <0.001) as depicted in Fig. 2b, however, State IV^+^ increased under high *P*CO_2_ at 8 °C compared to the group incubated under control *P*CO_2_ at the same temperature (*p* = 0.02). Fractional values of State IV^+^ in OXPHOS (Fig. 3) for the groups incubated under present levels of CO_2_ revealed values close to 20% in the groups incubated to 3 and 8 °C and two-fold higher values after incubation to 12 and 16 °C. In the groups incubated under high *P*CO_2_, State IV^+^ of the group incubated at 8 °C showed values similar to the groups incubated to 12 and 16 °C (Fig. 3). In consequence, sensitivity to CO_2_ varied with incubation temperature and was maximal but with opposite effects at 8 °C (stimulation of state IV^+^ above controls) and 16 °C (depression of OXPHOS below controls at 16 °C).

**Fig. 4:**
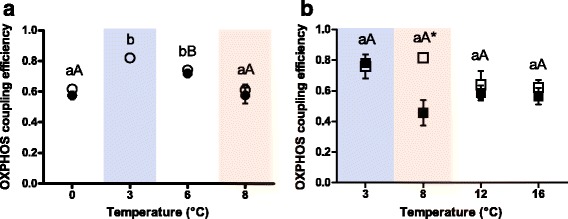
OXPHOS coupling efficiency in permeabilised heart muscle fibres of (**a**) Polar cod (*B. saida*) and (**b**) NEAC (*G. morhua*). Different letters within panels indicate significant differences (*p* <0.05) between temperature treatments; *lower case letters*: control *P*CO_2_ (400μatm), *upper case letters*: high *P*CO_2_ (1170μatm), * indicates significant differences (*p* <0.05) between CO_2_ groups at the same temperature. All values are reported as means ± S.E.M. (for *n* refer to Table 1). *Open symbols*: control *P*CO_2_ (400μatm), *filled symbols*: high *P*CO_2_ (1170μatm). *Circles*: Polar cod, *Squares*: NEAC. *Blue box*: cold shared incubation temperature (3 °C), *Red box*: warm shared incubation temperature (8 °C) between the two species

OXPHOS coupling efficiency in Polar cod under control *P*CO_2_ was maximal in the group incubated to 3 °C (0.82 ± 0.02), and decreased at 8 °C to values comparable to the 0 °C group (control *P*CO_2_: 0.61 ± 0.03, high *P*CO_2_: 0.58 ± 0.05), mainly because of increased State IV^+^ at 8 °C (Fig. 2, 3 and 4). In NEAC (Fig. 4b), the OXPHOS coupling efficiency was maximal at 8 °C and control *P*CO_2_ (0.81 ± 0.02) and minimal at 16 °C (0.64 ± 0.06). In the groups incubated under high *P*CO_2_, the maximum of OXPHOS coupling efficiency fell to 3 °C (0.77 ± 0.03) and reached its minimum at 8 °C (0.46 ± 0.08) to rise again at 12 °C and 16 °C (0.58 ± 0.05 and 0.56 ± 0.05, respectively). However, these changes in OXPHOS coupling efficiency were not significant (control *P*CO_2_: F = 5.27; *p* = 0.82, high *P*CO_2_: F = 9.7886, *p* = 0.072). At 8 °C, the OXPHOS coupling efficiency was significantly lower under high *P*CO_2_ than in the control *P*CO_2_ group (*p* = 0.003). Comparing the OXPHOS coupling efficiency between the two species, NEAC and Polar cod showed similar values in the 3 °C/control *P*CO_2_ group (Fig. 4 blue box) and at 8 °C/high *P*CO_2_ (*p* >0.05, Fig. 4 red box), while the coupling efficiency was higher in NEAC incubated at 8 °C/control *P*CO_2_ than in Polar cod incubated under the same conditions (*p* <0.001, Fig. 4 red box).

**Fig. 5:**
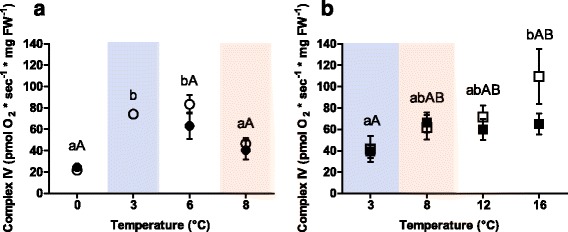
Complex IV (Cytochrome c Oxidase) capacity. Panel **a**: permeabilised heart muscle fibres of Polar cod (*B. saida*). Panel **b**: permeabilised heart muscle fibres of NEAC (G. *morhua*). Different letters within the panels indicate significant differences (*p* <0.05) between temperature treatments; *lower case letters*: control *P*CO_2_ (400μatm), *upper case letters*: high *P*CO_2_ (1170μatm). All values are reported as means ± S.E.M. (for *n* refer to Table 1). *Open symbols*: control *P*CO_2_ (400μatm), *filled symbols*: high *P*CO_2_ (1170μatm). *Circles*: Polar cod, *Squares*: NEAC. *Blue box*: cold shared incubation temperature (3 °C), *Red box*: warm shared incubation temperature (8 °C) between the two species

The thermal sensitivity of Complex IV also differed between the two species (Fig. 5). In Polar cod, Complex IV capacity rose from 0 to 6 °C (control *P*CO_2_: F = 67.29, *p* <0.001) and decreased between 6 °C and 8 °C (control *P*CO_2_: *p* <0.001). This trajectory was only present as a non-significant trend in the groups incubated under high *P*CO_2_ (F = 3.88, *p* = 0.10) because of the non-significant decrease of the mean capacity of Complex IV at 6 °C/high *P*CO_2_ compared to control *P*CO_2_ at the same temperature (*p* = 0.09). In NEAC, Complex IV capacity increased with increasing temperatures in the groups incubated under control *P*CO_2_ (F = 3.25, *p* = 0.05), but not in the groups incubated under high *P*CO_2_ (F = 2.18, *p* = 0.12). At 16 °C, the capacity of NEAC Complex IV was lower under high *P*CO_2_ (*p* =0.099) than under control *P*CO_2_. Comparing the two species, the capacity of Complex IV was similar (non-significant differences) in all shared treatments (3 °C/control CO_2_, 8 °C/control CO_2_ and 8 °C/high CO_2_: *p* >0.05, Fig. 5 blue and red boxes).

## Discussion

Our study shows differences in mitochondrial metabolism between a cold-adapted Arctic and a cold-acclimated sub-Arctic fish from the same area, potentially leading to differences in acclimation capacities to ocean acidification and warming.

Mitochondria from permeabilised heart fibres appeared to be affected mainly by the incubation temperature while high levels of CO_2_ significantly affected mitochondrial respiration only in NEAC (*Gadus morhua*) and mainly at the highest investigated temperature (16 °C). NEAC OXPHOS and Complex IV capacities decreased under elevated CO_2_ at high temperature, although the latter only as non-significant trend. This suggests that the noxious effects of high *P*CO_2_ are stronger at the upper end of the thermal window and might affect the heat tolerance of NEAC [2, 17]. Furthermore, proton leak at 8 °C was higher in the group incubated under high *P*CO_2_ than in the control *P*CO_2_ group, indicating that overall mitochondrial efficiency might be affected through alterations of membrane characteristics. Elevated *P*CO_2_ is reported to inhibit Citrate Synthase and Complex II in mammals and fish [40, 72, 73] with subsequent stimulation of the mitochondrial anaplerotic pathways to overcome this inhibition [40, 74]. The difference in sensitivity of the two species to elevated levels of CO_2_ could be related to differences in preferential metabolic pathways, with Polar cod (*Boreogadus saida*) relying more than NEAC on anaplerotic pathways that feed directly into Complex I such as the oxidation of glutamate, pyruvate or palmitoyl carnitine [40, 73]. Further investigation, especially at the genetic level is needed. Furthermore, it is still unknown whether and to what extent elevated *P*CO_2_ might alter the membrane characteristics and contribute to proton leak.

In Polar cod, OXPHOS of the groups incubated at 3-6-8 °C was higher at the respective incubation temperature than OXPHOS of the 0 °C treatments while the OXPHOS coupling efficiency was highest in the 3 °C group and lowest in the 0 and 8 °C groups. This indicates an optimum temperature for ATP production efficiency between 3 and 6 °C. At lower and higher temperatures, the increased proton leak in relation to OXPHOS created a less favourable ratio between ATP produced and oxygen consumed. These findings match those by Drost et al. [75], where heart rate of acutely warmed Polar cod increased until a first Arrhenius breakpoint at 3 °C. Heart rate still increased further but at a lower rate until 8 °C, passing a second break temperature. In our study, 8 °C corresponds to the highest rate of proton leak, and lowest Complex IV capacity, implying a direct participation of mitochondria in the thermal responses of the heart. The close similarity between the data from the acute study of Drost et al. [75], our 4-months incubation study and a study on behavioural thermal preference from Schurmann and Christiansen [76] indicates preferred temperatures of 3–6 °C within a thermal gradient from 0 to 8 °C for Polar cod, suggesting that Polar cod have only limited abilities to acclimate to higher temperatures.

In contrast, NEAC OXPHOS continued to increase with long-term incubation temperatures to even above those experienced within the natural habitat. This appears to occur without compromising OXPHOS coupling efficiency and reveals a higher acclimation potential than Polar cod, in line with the overall distribution area of Atlantic cod from temperate to (sub-) Arctic waters. This apparent plasticity is in line with the findings by Zittier et al. [77] in which NEAC specimens acclimated to 15 °C displayed critical temperatures (Tc, defined as the onset of the anaerobic metabolism, cf. Frederich & Pörtner [78]) about 10 °C higher than specimens kept at ambient temperature (4 °C). In Polar cod, the high proton leak at 8 °C is the main cause of reduced mitochondrial efficiency (OXPHOS coupling efficiency). This increase in proton leak can be caused by loss of membrane integrity in response to changes in membrane fluidity [7, 79]. In a previous study, Martinez et al. [80] found increased proton permeability of the inner mitochondrial membrane of the Antarctic silverfish *Pleuragramma antarcticum* after warming. In addition, Strobel et al. [40] found that this may be due to an unchanged saturation index of the mitochondrial membrane, observed in liver of the Antarctic *Notothenia rossii* after warm acclimation. These findings suggest a limited ability of Antarctic stenothermal fish to acclimate to temperature changes. Similar patterns may constrain acclimation of cold-adapted Arctic fish. The decreased capacity of Complex IV at 8 °C in Polar cod implies that the interactions between the inner membrane and embedded enzymes may also be affected by high temperatures [80, 81]. In NEAC, proton leak was lower than in Polar cod and reached 40% of OXPHOS at 12 °C, while in Polar cod the same relative values were found at 8 °C under control *P*CO_2._ A strong thermal response of proton leak may reflect high temperature sensitivity of the organism [52, 82–84], and thus a higher baseline proton leak combined with its steeper increase upon warming may point towards a stronger degree of cold adaptation in Polar cod.

The findings in this study contrast earlier results obtained in isolated mitochondrial suspensions where mitochondria remained fully functional beyond whole organism heat limits [82, 85]. The present findings suggest that mitochondria may display wider thermal limits in suspensions than when embedded in permeabilised fibres. Mitochondria in permeabilised fibres may still interact with other cellular organelles and are thus integrated into a more complex system than are isolated mitochondria. These considerations suggest that thermal tolerance is more constrained in permeabilized fibres than in isolated mitochondria. Such findings may thus be in line with the assumed narrowing of thermal windows once molecular and mitochondrial functions are integrated into larger units up to whole organism [86]. While the experiment was carried out at non-limiting *P*O_2_ in the media (>100 nmol ml^−1^) [87], diffusion gradients of oxygen and/or other substances within the permeabilised cardiac fibres may cause this hierarchy in thermal constraints. In a study on growth, mortality and standard metabolic rates (SMR) of the same Polar cod and NEAC as examined in this study, Kunz et al. [61] found higher SMR in Polar cod than in NEAC at the same incubation temperatures. This is mirrored in the mitochondrial respiration presented in this study, where OXPHOS capacity in Polar cod was larger than in NEAC at both 3 and 8 °C. In Polar cod, the SMR of the 3 and 6 °C groups were lower than in the groups incubated at 8 °C, which is mirrored in the pattern of cardiac State IV^+^ respiration. At 8 °C the OXPHOS coupling efficiency (i.e. ATP production efficiency) decreased as State IV^+^ increased and the capacity of Complex IV decreased. Maybe these findings indicate decreased cardiac mitochondrial efficiency that may limit cardiac function and promote heart failure, which is consistent with a drop in cardiac function [75] and the onset of heart failure in Polar cod at 8 °C. At this temperature, oxygen demand and mortality increased, and growth decreased in this species [61]. In fact, the estimated decrease in ATP production efficiency at 8 °C was paralleled by a reduced feed conversion efficiency and concomitant increase in SMR. This likely indicates a shift in energy allocation due to an impaired balance between energy production and demand, e.g. due to increased mitochondrial proton leak (see [88] for review). According to these findings, 8 °C is close to the long-term upper thermal tolerance limit for the Svalbard population of Polar cod, which is again in line with the observed increased mortality [61].

In NEAC, the parallel rise of whole organism SMR and cardiac fibre OXPHOS and the parallel decrease of OXPHOS and SMR at high *P*CO_2_ compared to controls at 16 °C indicates that cardiac mitochondrial function is adjusted to the level of whole animal energy demand at different incubation temperatures and that the effects of high *P*CO_2_ are greatest close to the upper thermal limit. Thermal constraints setting in at whole animal level may again relate to the thermal sensitivity of cardiac mitochondrial function [28, 29, 32, 33]. The fact that first performance limitations are observed in the 16 °C/high PCO_2_ incubation may not be of direct relevance for the Svalbard stock of NEAC over the next century, but marks a potential southern distribution limit for the Barents Sea and Norwegian Sea.

Polar cod is a cold adapted species and the constraint on cardiac mitochondrial metabolism at 8 °C, concomitant with increased mortality indicates that the animal’s thermal window matches its current habitat temperature range. In contrast, adult NEAC show the ability to broaden their thermal window beyond the present sub-Arctic habitat temperatures (see above). Because of the habitat temperature range of the two species is similarly wide but shifted to lower temperatures in Polar cod, combined with the high metabolic baseline cost (SMR) of Polar cod the two species may be classified as cold-adapted (Polar cod) or cold-acclimated (NEAC) eurytherms. NEAC appear to be much more plastic than Polar cod, thus, Polar cod may be more vulnerable to future ocean conditions than NEAC.

## Conclusions

Future ocean acidification and warming may impair cardiac mitochondrial function of Polar cod (*Boreogadus saida*) and Northeast Arctic cod (NEAC, *Gadus morhua*) in somewhat different ways. In Polar cod, high temperature (8 °C) increases proton leak and thereby decreases ATP production efficiency, while high CO_2_ levels did not have a significant effect. In NEAC, mitochondrial respiration remained functional at higher temperatures, but capacity was depressed by the combination of high temperature and high *P*CO_2_. Furthermore, in NEAC, incubation temperature leads to variable mitochondrial response patterns under elevated *P*CO_2_. The causes of the different responses to elevated *P*CO_2_ in the heart of these two species remain to be identified, for example, the role of anaplerotic pathways and their regulation should be further investigated.

As a result of the degree of cold adaptation, Polar cod display high metabolic maintenance costs (indicating that it is cold-eurythermal) and low acclimation capacity, while NEAC is cold acclimated and benefits from a lower rate of metabolism and a higher plasticity to acclimate to increasing temperature. As a consequence, mitochondrial function of NEAC hearts may be less constrained by rising temperatures than Polar cod, indicating that NEAC could outperform and possibly replace Polar cod in the waters around Svalbard if ocean warming and acidification further increase towards the conditions predicted for the end of the century (8 °C and 1170μatm *P*CO_2_). Since Polar cod has a key role in Arctic ecosystems [48], temperature driven changes in the distribution of this species can be an important component in the impacts of climate change on Arctic ocean ecosystems.
